# Mutations Designed by Ensemble Defect to Misfold Conserved RNA Structures of Influenza A Segments 7 and 8 Affect Splicing and Attenuate Viral Replication in Cell Culture

**DOI:** 10.1371/journal.pone.0156906

**Published:** 2016-06-07

**Authors:** Tian Jiang, Aitor Nogales, Steven F Baker, Luis Martinez-Sobrido, Douglas H Turner

**Affiliations:** 1 Department of Chemistry, University of Rochester, Rochester, New York, United States of America; 2 Department of Microbiology and Immunology, University of Rochester, Rochester, New York, United States of America; German Primate Center, GERMANY

## Abstract

Influenza A virus is a significant public health threat, but little is understood about the viral RNA structure and function. Current vaccines and therapeutic options to control influenza A virus infections are mostly protein-centric and of limited effectiveness. Here, we report using an ensemble defect approach to design mutations to misfold regions of conserved mRNA structures in influenza A virus segments 7 and 8. Influenza A mutant viruses inhibit pre-mRNA splicing and attenuate viral replication in cell culture, thus providing evidence for functions of the targeted regions. Targeting these influenza A viral RNA regions provides new possibilities for designing vaccines and therapeutics against this important human respiratory pathogen. The results also demonstrate that the ensemble defect approach is an efficient way to test for function of RNA sequences.

## Introduction

Influenza A virus is a segmented, single-stranded, negative sense RNA virus [1]. Every year, 15–20% of the world’s population is infected, and 250 to 500 thousand people are killed by influenza A virus [2]. Currently, the most widely used vaccine is the Inactivated Influenza Vaccine (IIV), which is given by intramuscular injection [3]. IIV is noninfectious, and thus recommended for all people above 6 months of age [4], but its immunogenicity is relatively weak [5, 6]. The other option for vaccination is the Live-Attenuated Influenza Vaccine (LAIV), which is given intranasally [7]. The live-attenuated virus includes five mutations within the A/Ann Arbor/6/60 H2N2 backbone [8, 9]. LAIV is temperature sensitive, so it only replicates in the cooler upper respiratory tract (33°C), but cannot damage the warmer lower respiratory tract (37°C). Because of safety concerns, it is only recommended for healthy, non-pregnant individuals 2–49 years of age [10, 11]. In the 2014–2015 influenza season, the overall vaccine effectiveness was as low as 19% [12], due to antigenic drifts in circulating H3N2 viruses. There is a huge demand for increasing both safety and protective efficacy of current influenza vaccine approaches. Structure targeted mutations in mRNA can potentially be used as a new approach to develop weakened virus for LAIV.

Two classes of therapeutics have been approved by the Food and Drug Administration (FDA) for use against influenza: adamantanes and neuraminidase inhibitors [13]. Both target essential proteins coded by influenza, the ion channel protein M2, and the neuraminidase protein, respectively [14]. Most circulating influenza viruses are already resistant to adamantanes [15, 16], and are starting to build up resistance to neuraminidase inhibitors [17]. No new anti-influenza drug has been approved in the US since 1999. Most new targets being investigated are still protein-centric [13]. Conserved viral RNA structures important for function are potentially new therapeutic targets.

**Fig 1:**
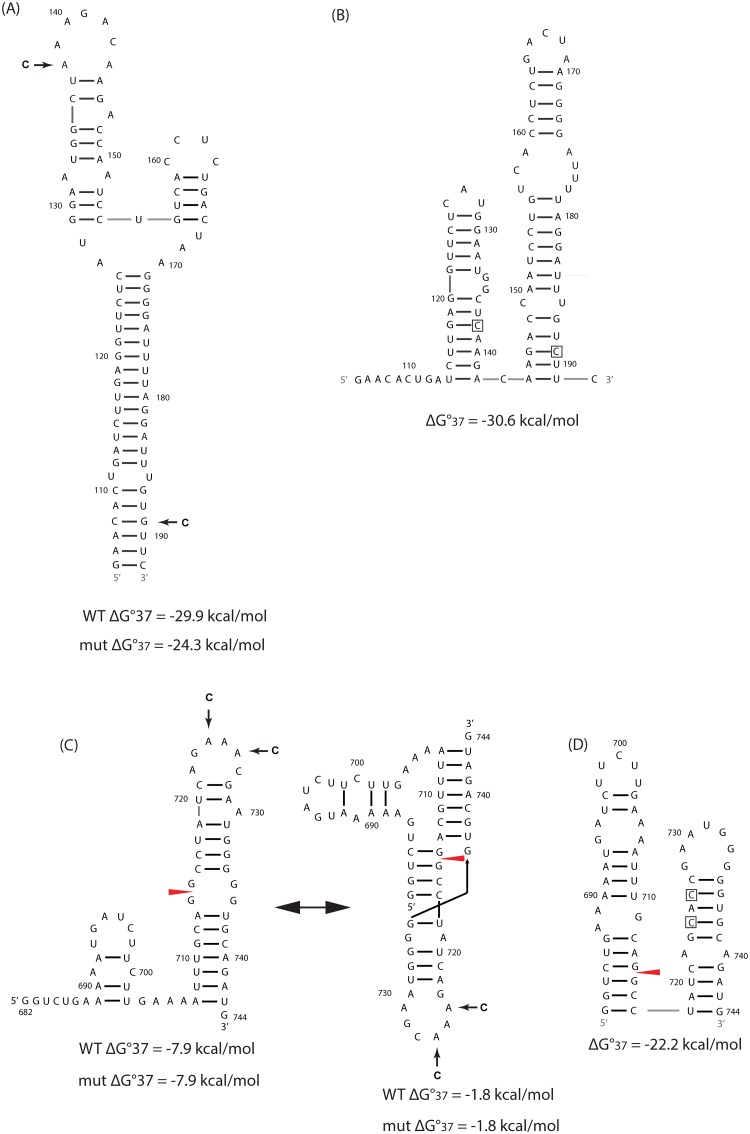
Conserved RNA structures identified in mRNAs of influenza A virus segment 7 and predicted folding of ensemble defect mutants. Ensemble defect designed mutations are indicated by arrows in A and C and by boxes in B and D. Wild-type (WT) sequences are from influenza A/Puerto Rico/1934 H1N1 (PR8) strain. Predicted free energies of folding at 37°C for WT and mutated sequences are provided below each structure. For hairpins and pseudoknots, respectively, ΔG°_37_ was predicted by RNAstructure [26] and ShapeKnots programs [27]. (A) The three-way multi-branch loop, 7MB, 79 nts downstream from the 5′ splice site in segment 7 [20]. (B) The predicted folding of 7MB_ED. (C) Pseudoknot/ hairpin equilibrium at the 3′ splice site in segment 7 [21]. (D) The predicted folding of 7PK_ED.

**Fig 2:**
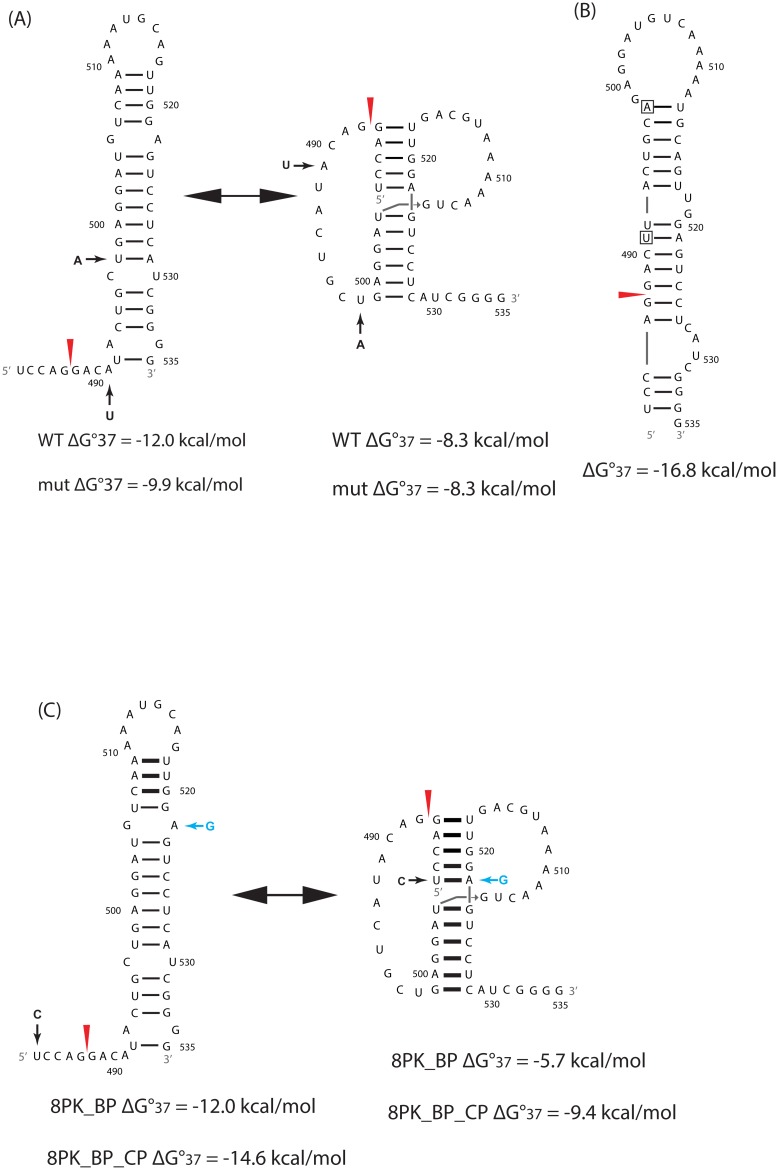
Conserved RNA structures identified in mRNAs of influenza A virus segment 8 and predicted folding of ensemble defect mutants. (A) Pseudoknot/ hairpin equilibrium at the 3′ splice site in segment 8 [19]. The lower helix in the pseudoknot conformation may be extended by 4 additional base pairs and a single mismatch forming between nts 494–498 and 529–533 [19]. (B) The predicted folding of 8PK_ED. (C) Black arrow corresponds to mutation in 8PK_BP and blue arrow corresponds to compensating mutation in 8PK_BP_CP. Details are same as in Fig 1 caption.

Segments 7 and 8 of influenza A virus code for M1/M2 and NS1/NEP proteins, respectively [1], where M1 is a matrix protein connecting vRNAs to each other and to the viral envelope, NS1 is an interferon antagonist, and NEP is a nuclear export protein. Based largely on sequence comparison and predicted thermodynamics, conserved RNA structures were identified near or containing splice sites in segments 7 and 8 mRNAs [18, 19]. In segment 7, a three-way multi-branch loop (7MB) that is 79 nucleotides (nts) downstream from the 5′ splice site [20] (Fig 1A) and a pseudoknot/hairpin equilibrium (7PK) at the 3′ splice site [21] (Fig 1C) were confirmed *in vitro* by enzymatic and chemical mapping. In segment 8, a pseudoknot/hairpin equilibrium (8PK) at the 3′ splice site [19] (Fig 2A) was identified. In this work, each of these conserved structures was mutated by changing only two nucleotides predicted by an ensemble defect program [22] to completely change the secondary structure. In cell culture, the influenza A mutants affected mRNA splicing and attenuated viral replication. Similar effects were also observed upon disrupting a single UA base pair in a pseudoknot containing the 3′ splice site of segment 8. The results demonstrate that the ensemble defect approach can rapidly reveal function for regions predicted to fold into stable secondary structures.

## Experimental Methods

### Cells

Madin-Darby canine kidney cells (MDCK; ATCC CCL-34), human lung epithelial carcinoma cells (A549; ATCC CCL-185), and human embryonic kidney 293T cells (293T; ATCC CCL-11268) were maintained in Dulbecco’s modified Eagle’s medium (DMEM; Mediatech, Inc.) supplemented with 10% fetal bovine serum (FBS; Atlanta Biologicals) and 1% PSG (penicillin, 100 units/ml; streptomycin, 100 μg/ml; L-glutamine 2 mM; Mediatech, Inc.). Cells were grown at 37°C in air enriched with 5% CO_2_.

### Design of mutations using an ensemble defect program

The ensemble defect program was adapted from *Zadeh*, *et al*. [22]. Ensemble defect, n, is used here to choose a sequence, Φ, unlikely to fold into the wild type (WT) secondary structure, s. Ensemble defect uses thermodynamics to predict the average number of nucleotides paired differently in mutated sequence, Φ, relative to WT structure, s, at equilibrium over the ensemble of possible secondary structures:

$$n\left(\emptyset ,s\right)=\sum_{\sigma \in \Gamma }p\left(\emptyset ,\sigma \right)d\left(\sigma ,s\right)$$

Here, Γ corresponds to the unpseudoknotted structural ensemble for the sequence Φ. p(Φ, σ) is the equilibrium probability of any secondary structure σ belonging to Γ (i.e. a secondary structure that can be formed by the sequence Φ) calculated using the partition function [23, 24]. d(σ, s) is the distance between the WT secondary structure, s, and another secondary structure, σ, with d defined as the number of nucleotides paired differently in the two structures. Thus, the goal is to computationally maximize n to predict a sequence, Φ, that will fold into a new secondary structure that is very different and much more probable than the WT structure.

For the target sequences, all 2-nt mutations were computed and used to calculate the ensemble defects. Sequences with a maximized ensemble defect and minimal amino acid changes were manually selected. For the WT sequences that can fold into an equilibrium between a hairpin and a pseudoknot, the ensemble defects were calculated against the hairpin conformation. The free energy of folding at 37°C, ΔG°_37_, was predicted by the RNAstructure [25] and the ShapeKnots program [26] for unpseudoknotted and pseudoknotted structures, respectively.

### Site-directed Mutagenesis

QuickChange Site-Directed Mutagenesis kit (Agilent Technologies) was used to introduce mutations in pPolI M and NS plasmids [27]. Briefly, PCR reactions were assembled with two oligonucleotide primers containing the desired mutations, following manufacturer’s recommendations. The oligonucleotide primers, each complementary to opposite strands of the M and NS viral segments, were extended during temperature cycling by the *Pfu*Turbo DNA polymerase. Incorporation of the primers generates a plasmid containing the desired mutations. Following temperature cycling, Dpn I was added to the reaction mix and the sample was incubated for 1 h at 37°C to digest the parental plasmids, leaving only the non-methylated plasmids generated by PCR. The resulting plasmids containing the desired mutations were then transformed into the XL1-Blue supercompetent cells. To ensure correct mutations, plasmids were sequenced (Genewiz, Inc.).

### Generation of influenza viruses from plasmid DNA

WT influenza A/Puerto Rico/8/1934 H1N1 (PR8) virus [27] was generated using plasmid-based reverse genetic techniques, as described previously [28]. Briefly, co-cultures (1:1) of MDCK/293T cells (6-well plate format, 10^6^ cells/well) were co-transfected with ambisense plasmids (pDZ) containing the eight PR8 viral segments using Lipofectamine 2000 (Invitrogen). At 16 h post-transfection, the medium was changed to DMEM containing 0.3% bovine serum albumin (BSA), 1% PSG, and 1 μg/ml tosylsulfonyl phenylalanyl chloromethyl ketone (TPCK)-treated trypsin (Sigma). At 48 h post-transfection, tissue culture supernatants (TCS) were collected and used to infect fresh MDCK cells. At 48 h post-infection, recombinant viruses were plaque purified in MDCK cells. Virus stocks were generated by infecting confluent 10-cm dishes of MDCK cells at a multiplicity of infection (MOI) equal to 0.001. Viruses containing designed mutations were generated similarly using the pPolI M or NS plasmids containing the desired mutations instead of the WT pDZ plasmids. After preparing the mutant virus stocks, the mutated genomic segment was amplified by RT-PCR and sequenced to ensure the presence of introduced mutations.

### Determination of virus titers using immunofluorescence assay (IFA)

Influenza PR8 WT and mutant viral titers were measured by immunofocus assay as described by Baker, et al. [29]. Briefly, 10-fold serial dilutions of viruses were used to infect confluent monolayers of MDCK cells (10^4^ cells/well, 96-well plate format) in triplicate. Cells were incubated with viruses for 1 h at room temperature to allow virus absorption. After that, cells were washed with PBS, and incubated with DMEM containing 0.3% BSA, 1% PSG, and 1 μg/ml TPCK-treated trypsin at 37°C for 8 h. Then cells were fixed and permeabilized in 4% formaldehyde and 0.5% Triton X-100 for 15 min at room temperature. After washing with PBS, the cells were incubated in the blocking solution of 2.5% BSA overnight at 4°C, and then incubated with 1 μg/ml of an anti-influenza A NP antibody (HT103) for 1 h at 37°C. After washing three times with PBS, the cells were incubated with a fluorescein isothiocyanate (FITC)-conjugated rabbit anti-mouse IgG secondary antibody (Dako) and 1 mg/ml of 4’,6’-diamidino-2-phenylindole (DAPI; Research Organics) for 1 h at 37°C. Cells were visualized under a fluorescence microscope, and fluorescently-labeled NP-expressing cells were quantified to determine the virus titer (fluorescent focus forming units [FFU]/ml). The mean value and standard deviation were calculated using Microsoft Excel software.

### Virus multi-cycle growth kinetics

Multi-cycle viral growth kinetics were measured in MDCK and A549 cells (5 x 10^5^ cells/well, 12-well plate format, triplicates) by infecting with WT or mutant viruses (MOI of 0.001 or 0.0001). After virus absorption for 1 h at room temperature, cells were washed with PBS, overlaid with DMEM containing 0.3% BSA, 1% PSG, and 1 μg/ml TPCK-treated trypsin, and incubated at 37°C. TCS were collected at indicated time points post-infection, and virus titers were measured using the immunofocus assay, as described above. Mean value and standard deviation (SD) were calculated using Microsoft Excel software.

### Quantitative RT-PCR (qRT-PCR)

WT and mutant viruses were used to infect MDCK or A549 cells (5 x 10^5^ cells/well, 12-well plate format, triplicates) with MOI of 5. An MOI of 5 was required to generate enough viral RNA for qRT-PCR. Cells were collected at 3 h post-infection, and total RNAs were extracted using RNeasy kit (Qiagen). One μg total RNA was reverse transcribed using oligo(dT) primers with SuperScript II reverse transcriptase (Invitrogen) to amplify mRNA. StepOnePlus Real-Time PCR System and Fast SYBR Green Master Mix (Invitrogen) were used for qPCR with the cDNAs as templates. Primers used for amplification are listed in S1 Table. The relative mRNA levels were analyzed using the Pfaffl method [30]. Amplification specificity was confirmed by melting curve analysis at the end of each program.

## Results

### Mutations designed to misfold three conserved RNA structures by maximizing ensemble defects

An ensemble defect program (see Methods) was used to predict sequences with nucleotides paired completely differently from the WT sequence. For three conserved RNA structures from segments 7 and 8, separate 2-nt mutations, 7MB_ED, 7PK_ED, and 8PK_ED, were predicted to maximize ensemble defect (Figs 1, 2A and 2B). Thus, the mutated sequences are forced to fold predominately into secondary structures different from WT.

In 7MB_ED and 7PK_ED, the mutants are predicted to fold into double hairpin conformations (Fig 1B and 1D). The free energy changes for folding at 37°C are more favorable by 6.3, 14.3, and 20.4 kcal/mole, respectively, compared with the WT structure of the multi-branch loop, the hairpin, and pseudoknot conformations if formed by the mutated sequences at 37°C. (Note that at 37°C, every change by 1.4 kcal/mol corresponds to a 10-fold change in equilibrium constant for folding.)

The mutations in 7MB_ED (A138C and G189C) are silent. The mutations in 7PK_ED (A724C and A726C) are 3′ from the splice site, and change three amino acids: K242H in M1, and R12S plus N13T in M2 (Table 1). The same nucleotide and amino acid mutations exist in a natural strain, A/duck/Jiangsu/2012 (H5N2) [31].

**Table 1:** Naturally occurring counts of WT and corresponding mutated amino acids for the listed mutant PR8 viruses. Mutations in 7MB_ED and 8PK_BP are silent. All sequences were downloaded from the National Center for Biotechnology Information (NCBI) resource. Unique sequences were extracted from the alignment, and the total number of unique sequences is listed in the last column.

mutant	protein	amino acid position	wild type sequence	occurrence	mutation	occurrence	total seqs
**7PK_ED**	**M1**	242	K	164	H	1	201
**7PK_ED**	**M2**	12	R	245	S	1	302
**7PK_ED**	**M2**	13	N	149	T	39	302
**8PK_ED**	**NS1**	169	H	402	L	2	421
**8PK_ED**	**NEP**	12	I	263	L	1	280
**8PK_ED**	NEP	14	L	26	Q	24	280
**8PK_BP_CP**	**NEP**	22	E	52	G	140	280

In 8PK_ED, the mutations (A491U and U498A) enable the mutant to fold into a single hairpin conformation different from WT (Fig 2A and 2B). The predicted free energy is more favorable by 6.9 and 8.5 kcal/mole, respectively, compared with the WT hairpin and pseudoknot conformations if formed by the mutated sequence at 37°C. The mutations are 3′ from the splice site, and change three amino acids: H169L in NS1, and I12L and L14Q in NEP (Table 1). That combination of amino acid changes has not been observed in natural influenza A strains.

NMR spectra for the 3′splice site pseudoknot in segment 8 of strain A/duck/Shanghai/13/01 [16] revealed coaxial stacking between the two helixes [19]. On the basis of sequence comparison, two mutants, 8PK_BP and 8PK_BP_CP, were designed to, respectively, disrupt and repair the coaxial stack (Fig 2C). Mutant 8PK_BP has a single silent mutation, U483C, that destabilizes the pseudoknot conformation by 2.6 (= 8.3–5.7) kcal/mol at 37°C (Fig 2A and 2C), while the hairpin conformation is predicted to maintain the same thermodynamic stability. The compensatory mutation, A522G, in 8PK_BP_CP restores the canonical base pair between nt 483 and nt 522, thus allowing coaxial stacking (Fig 2C). A522G changes an amino acid, E22G, in NEP. Both E and G exist widely at position 522 (Table 1). A single A522G mutation was not tested because in the pseudoknot, this would replace an AU pair with a GU pair, which is predicted to destabilize the helix by only 0.1 kcal/mol at 37°C [32]. Similarly, in the hairpin conformation, a GA pair would be replaced with a GG pair, which is predicted not to change folding.

### Mutated viruses affect splicing of segments 7 and 8 pre-mRNAs in cell culture

**Fig 3:**
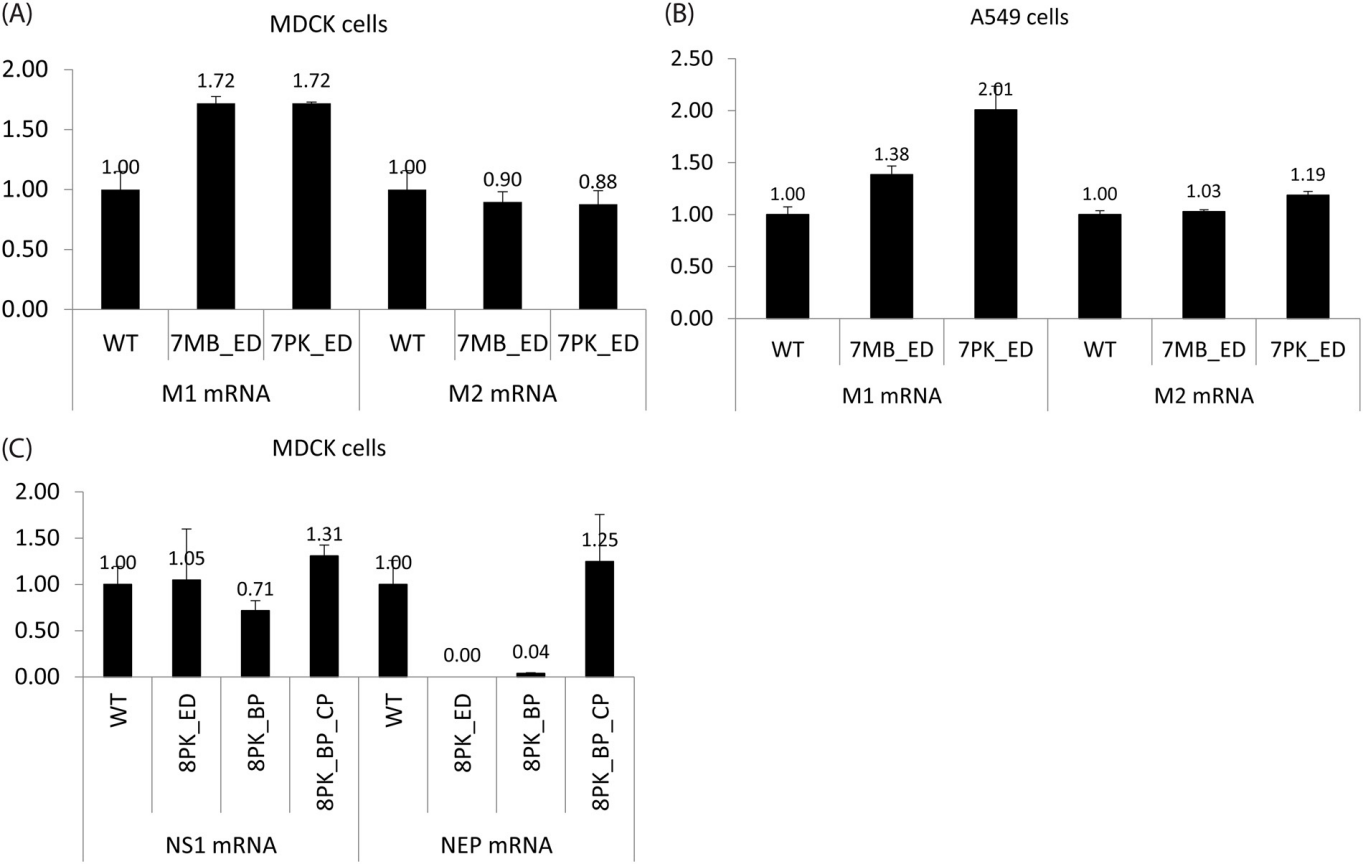
The relative abundance of unspliced and spliced mRNAs of WT and mutant viruses. Total RNAs were collected 3 h post-infection (MOI = 5). The mRNA relative abundance was quantified by qRT-PCR, and normalized to the WT virus. 7MB_ED and 7PK_ED increased the amount of M1 mRNA in (A) MDCK cells and (B) A549 cells. (C) 8PK_ED and 8PK_BP decreased the amount of NEP mRNA in MDCK cells, while 8PK_BP_CP restored the splicing level.

Plasmid-based reverse genetics techniques [28] were used to generate the recombinant mutated segment 7 and 8 PR8 viruses. All the mutant viruses were successfully rescued with titers similar to those of WT PR8. Sequencing showed that they retained the designed mutations (data not shown). For segment 7 mutants, total RNAs were extracted from MDCK and A549 cells 3 h after infection (MOI = 5) at 37°C. MDCK cells were used because they are common models for influenza studies [33]. Human lung epithelial A549 cells were used because they are more representative of cells infected in humans. The relative amounts of M1 and M2 mRNAs were measured using qRT-PCR. The results showed that M2 mRNA abundance was similar in WT and mutant viruses, while M1 mRNA abundance was raised about 1.4 to 2 fold in the mutant viruses as compared to WT (Fig 3A and 3B). Similar results were observed for both MDCK and A549 cells.

For segment 8 mutants, total RNAs were extracted from MDCK cells 3 h after infection (MOI = 5) at 37°C with WT, 8PK_ED, 8PK_BP, and 8PK_BP_CP. Measurements by qRT-PCR on 8PK_ED and 8PK_BP revealed that the relative amount of NEP mRNA was dramatically reduced as compared with WT, and even undetectable in 8PK_ED (Fig 3C). With the compensating base pair in 8PK_BP_CP, the lost NEP mRNA was restored to a level comparable with WT. The relative amount of the unspliced product, NS1 mRNA, was similar in WT and the mutant viruses (Fig 3C).

### Mutations attenuate virus replication in cell culture

**Fig 4:**
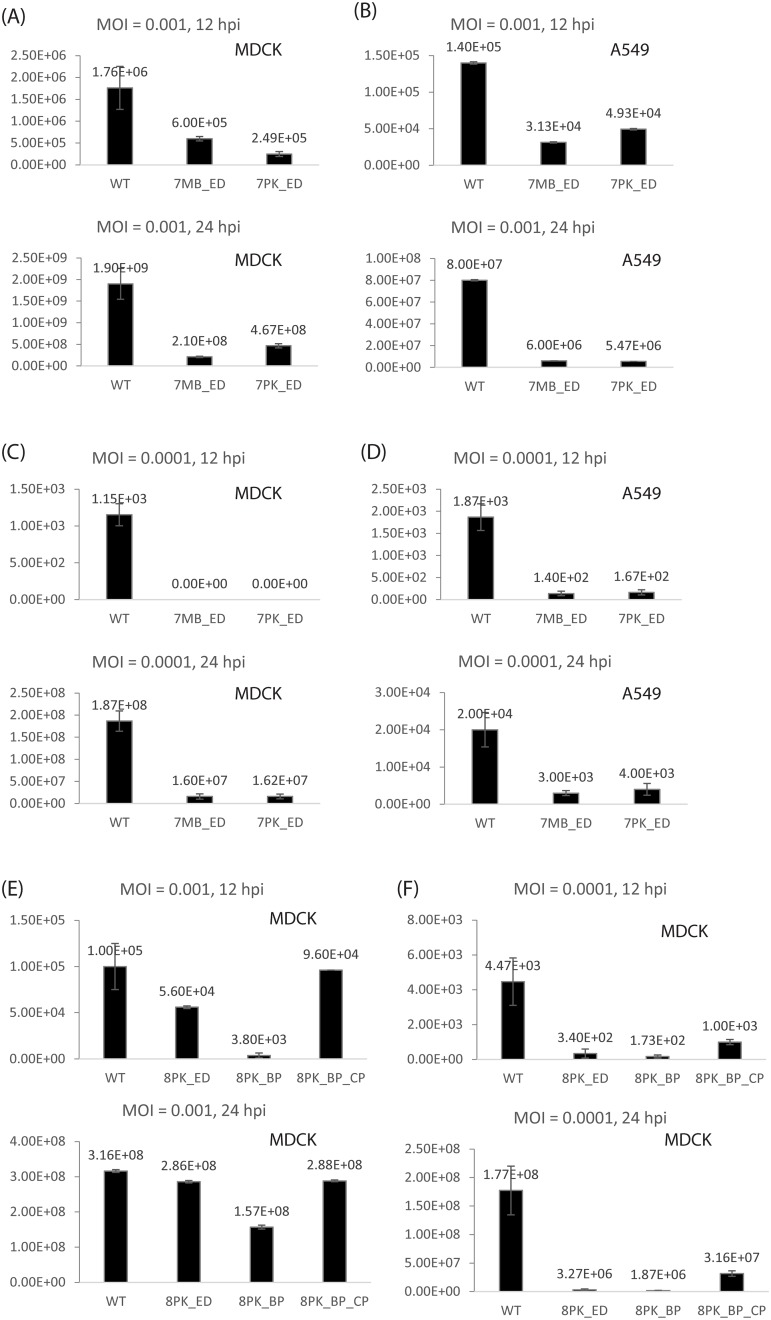
Viral replication was attenuated at 12 and 24 h post-infection in MDCK cells and A549 cells with MOI = 0.001 or 0.0001 at 37°C. Virus titers were quantified by immunofocus assay (FFU/ml). Data represent the means ± SDs of the results determined for triplicate wells. Replication of WT, 7MB_ED, and 7PK_ED were examined in (A) MDCK cells and (B) A549 cells with MOI = 0.001, and in (C) MDCK cells and (D) A549 cells with MOI = 0.0001. Replication of WT, 8PK_ED, 8PK_BP, and 8PK_BP_CP were examined in MDCK cells with (E) MOI = 0.001 and (F) MOI = 0.0001.

Multi-cycle growth kinetics at 37°C of WT and mutant viruses were measured in MDCK cells. WT and segment 7 mutants were also examined in A549 cells (Fig 4). Using MOI = 0.001, viruses with either 7MB_ED or 7PK_ED showed attenuated kinetics of replication in both cell lines, ranging from about 3–15 fold compared with WT virus at 12 and 24 h post-infection (Fig 4A and 4B). 8PK_BP mutation attenuated viral replication by about 26-fold in MDCK cells at 12 h post-infection, while 8PK_BP_CP and 8PK_ED showed similar growth kinetics as WT PR8 (Fig 4E).

With MOI = 0.0001, attenuation of the mutants was more dramatic. In MDCK cells, the viral titers of 7MB_ED and 7PK_ED were below detectable levels (20 FFU/ml) at 12 h post-infection, at least 58-fold attenuated relative to WT PR8 (Fig 4C). The differences were smaller in A549 cells, but were still more than 10-fold at 12 h post-infection (Fig 4D). 8PK_BP and 8PK_ED were also attenuated with MOI = 0.0001, ranging from about 13–95 fold compared with WT virus at 12 or 24 h post-infection (Fig 4F). The attenuation with 8PK_BP was partially reversed by the compensatory mutant, 8PK_BP_CP, which replaces a UA pair with a CG pair expected to make coaxial stacking between two helixes [19] more favorable [34–36]. All the mutated viruses were sequenced at 72 h post-infection. No revertants or additional mutations were observed (data not shown).

## Discussion

Little is known about the biological function of RNA structure in influenza A virus [37], and no current vaccines or therapeutics are designed to target viral RNA structures. Conserved RNA secondary structures throughout protein coding regions of influenza A mRNAs were predicted by bioinformatics using a combination of thermodynamics and comparative sequence analysis, including suppression of synonymous codon usage [18, 19]. Structures predicted in mRNAs of segments 7 and 8 (Figs 1A, 1C and 2A) are most exciting because they locate near or containing splice sites. This suggests that these conserved RNA structures may be important for the splicing of segments 7 and 8 mRNAs. Enzymatic and chemical mapping, and nuclear magnetic resonance (NMR) provided experimental evidence for the predicted RNA structures [19–21]. In this work, mutational evidence is provided that is consistent with biological function for three of these structures. Sequences with only one or two mutations out of the 13.5 kb genome inhibited viral mRNA splicing and attenuated viral replication in cell culture. Evidently, the ensemble defect approach provides an efficient method to test for function of conserved structures.

### The multi-branch loop and pseudoknot/hairpin region in segment 7 mRNA have function

An ensemble defect program [22] was used to design constructs, 7MB_ED and 7PK_ED, with 2-nt mutations predicted to misfold the multi-branch loop and pseudoknot/hairpin regions, respectively, in segment 7 mRNA (Fig 1). Both mutants increased the relative abundance of M1 mRNA compared with WT, while M2 mRNA levels remained the same (Fig 3A and 3B). This suggests that these conserved RNA structures affect the efficiency of segment 7 splicing, but a feedback loop regulates the amount of spliced mRNA. When production of M2 mRNA is inhibited, the virus may produce more M1 mRNA to ensure that enough M2 mRNA and thus enough essential M2 protein is produced. Both mutants also similarly reduced viral titers in both MDCK and A549 cells, with results most dramatic at 12 h post-infection at the lower MOI of 0.0001 (Fig 4A–4D).

It is noteworthy that 7MB_ED and 7PK_ED have similar effects on M1 and M2 mRNA levels, and on viral replication. The mutations in 7MB_ED are silent, whereas those in 7PK_ED change two amino acids in M2 protein and one in M1 protein (Table 1). The results suggest both mutants affect splicing to a similar level regardless of amino acid changes. The changes in conserved RNA structures are most likely responsible for the observed phenotype. Thus, the identified multi-branch loop near the 5′ splice site and the pseudoknot/hairpin equilibrium at the 3′ splice site apparently play a role in the regulation of mRNA splicing.

### The pseudoknot/hairpin region in segment 8 has function

Two mutants were constructed to change the structure of 8PK (Fig 2), and both exhibited a similar phenotype. The 8PK_ED (Fig 2A and 2B) and 8PK_BP (Fig 2C) mutants greatly reduced the amount of spliced mRNA (Fig 3C). At 37°C, using MOI = 0.001, 8PK_ED showed similar growth kinetics as WT virus, while 8PK_BP was attenuated at 12 h post-infection (Fig 4E). At 37°C with MOI = 0.0001, the replication of both 8PK_ED and 8PK_BP were attenuated by 13–95 fold at 12 and 24 h post-infection (Fig 4F). 8PK_BP_CP, which compensated the mutation in 8PK_BP, restored WT splicing, and increased the viral titers relative to 8PK_BP. With MOI = 0.001, the viral titer of 8PK_BP_CP was increased to a level comparable with WT while with MOI = 0.0001, the viral titer of 8PK_BP_CP relative to 8PK_BP was increased by about 6 and 17-fold at 12 and 24 h post-infection, respectively. Because segment 7 mutants have similar effects in MDCK and A549 cells, segment 8 mutants were only examined in MDCK cells.

The results suggest that the phenotypes observed with the mutants in segment 8 are due to changes in RNA structure. In the pseudoknot conformation, the U483-A522 pair is expected to be coaxially stacked with the adjacent U504-G523 pair [19]. 8PK_BP disrupts U483-A522, and thus the coaxial stacking, which may be important for regulating splicing. 8PK_BP_CP restores the observed phenotype because the mutant restores the base pairing between nt 483 and nt 522. A mutation that disrupts the canonical U483-A522 pair was observed in highly pathogenic H5N1 viruses, and caused the equilibrium to shift towards the hairpin conformation [19].

### Amino acid changes are not expected to have large effects

In 7PK_ED, 8PK_ED, and 8PK_BP_CP, the mutations of the mRNA sequences also produce local mutations in amino acid sequences. The naturally occurring counts of the WT and the corresponding mutated amino acids for these three mutants are summarized in Table 1.

**Fig 5:**
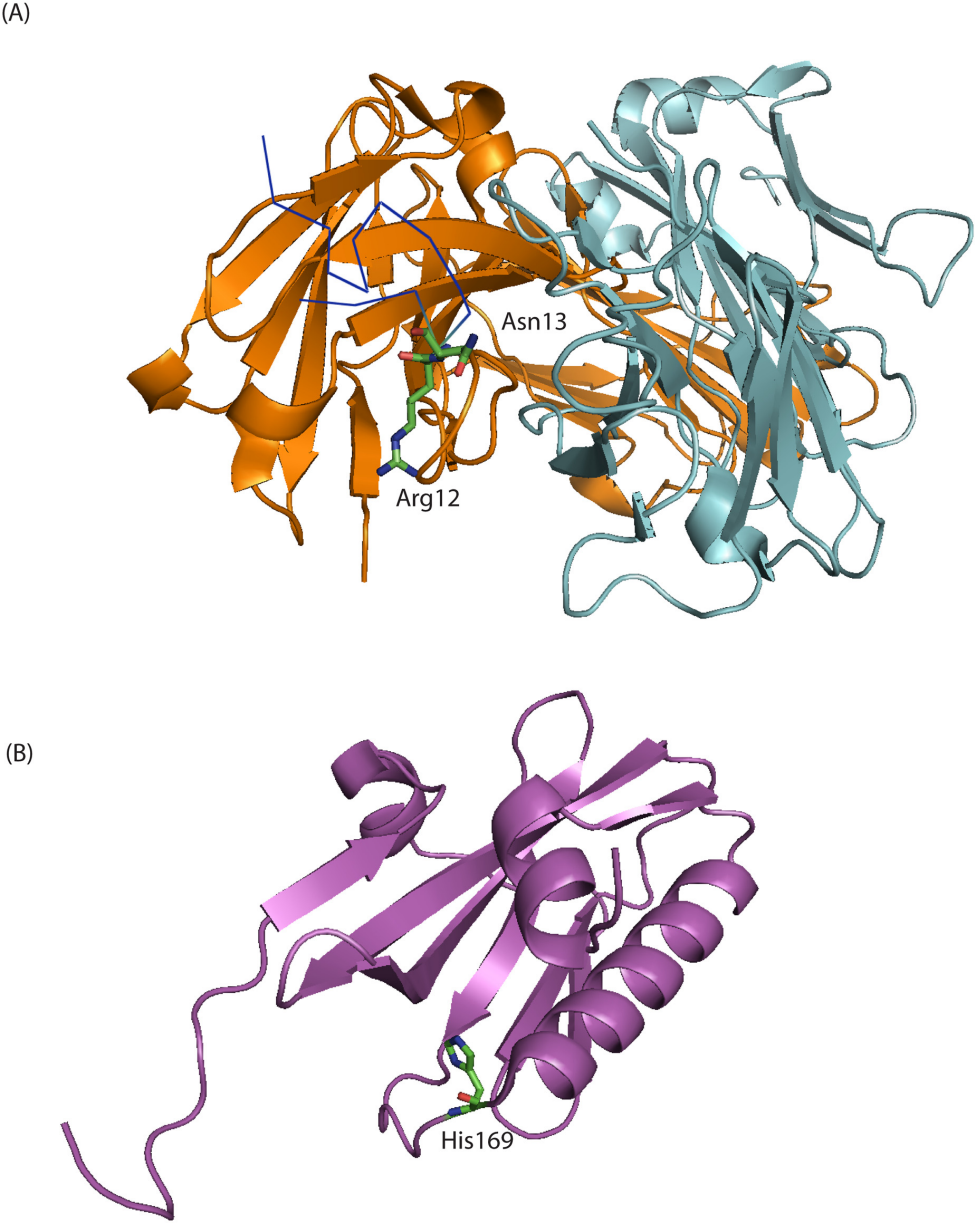
(A) 3D structure of influenza M2 ectodomain in complex with monoclonal antibody Fab65 [46]. PDB ID: 4N8C. The light chain (light blue) and heavy chain (orange) of Fab65 are shown in cartoon presentation. R12 and N13 are labeled and shown in stick presentation. 7PK_ED has mutations R12S and N13T. The other residues of M2 ectodomain (dark blue) are shown in ribbon presentation. (B) 3D structure of NS1 C-terminal region [47]. PDB ID: 2GX9. The NS1 monomer (magenta) is shown in cartoon presentation. H169 is labeled and shown in stick presentation. 8PK_ED has mutation H169L.

For 7PK and 8PK regions, the peptide motifs of M1, M2, and NEP corresponding to the conserved RNA structures are all in largely unstructured conformations [38–45], thus expected to be tolerant to mutations. The 7PK region corresponds to part of the C terminal domain of M1, which is known to be extended and partially flexible on the basis of circular dichroism and bioinformatics [39, 41–43]. The 7PK region also corresponds to part of the ectodomain of M2, which exists in a dynamic conformation [38, 44, 45]. The crystal structure of the M2 ectodomain in complex with a protective monoclonal antibody suggests that the M2 ectodomain may have different conformations, including a curled structure that can induce binding with specific antibodies [46]. Neither of the M2 WT amino acids, R12 and N13, mutated in 7PK_ED had direct contacts with the antibody, but they facilitated polar interactions between M2 and the antibody (Fig 5A). S12 and T13 in 7PK_ED may facilitate similar interactions. The similar phenotypes observed for 7PK_ED and the silent mutations in 7MB_ED (Figs 3A, 3B and 4A–4D) are consistent with the assumption that changes in protein sequence are not responsible for the phenotypes.

The 8PK region corresponds to the N terminal domain of the nuclear export protein, NEP, which exists in a highly mobile and exposed state [40]. This flexibility may be important for the recognition of substrate proteins by the nuclear export machinery. The peptide motifs of NS1 corresponding to the RNA sequences mutated in 8PK are in an α-helical region [47–51] (Fig 5B). H169 locates at the end of the α-helix, thus mutation at this position has minimal effects in changing the propensities of forming α-helices [52]. It would be surprising if the mutated amino acids make a strong contribution to the observed phenotypes, but this cannot be ruled out.

### Potential implications for developing safer vaccine

The temperature-sensitive (*ts*) and attenuated (*att*) phenotype of LAIV is imparted by five mutations within the viral replicative machinery: PB2 N265S; PB1 K391E, D581G and A661T; and NP D34G [8, 53]. Although this vaccine has an acceptable safety profile and is in general more effective than IIV, it is only recommended for use in healthy immunocompetent people because of possible complications in the very young and in patients with pre-existing conditions [54, 55].

The mutations identified in this work that attenuated virus replication were made in segments 7 and 8, rather than in the segments currently mutated in LAIV. This suggests such mutations could potentially be introduced to LAIV to further decrease the virulence of the vaccine, and thus increase its safety profile [56]. Also, these mutations have the potential to serve as replacements if influenza evolves revertants to some currently used mutations.

### Potential implications for developing new treatments

Because the conserved RNA structures are functionally important, oligonucleotides and small molecules targeting them may be designed as potential therapeutics against influenza A virus. For example, the M1 and M2 proteins coded by segment 7 are both essential, so inhibiting splicing is a potential therapeutic strategy. In general, a given mRNA or vRNA will produce many copies of protein, so therapeutic options targeting RNA structures would have to inhibit fewer copies of target than therapeutics targeting proteins.

### Refolding RNA structure by designing minimal mutations predicted to misfold structure by maximizing ensemble defects can be an efficient way to test regions of sequence for phenotype

This is the first time that a program maximizing ensemble defects [22] was used to design sequences to refold conserved RNA structures for functional studies. As few as 2-nt mutations were required to test for a phenotype. This method provides an alternative to the traditional methods that destabilize specific base pairs. The method may also be applicable to unstructured regions because tight folding of an RNA structure in a mutant could inhibit dynamics and/or protein binding. Thus, it opens new avenues for targeting RNA sequences and structures for the development of safer vaccines and of therapeutics to control infections.

## Supporting Information

S1 Fig Multi-cycle growth kinetics of WT and mutant viruses plotted in log-scale. (DOCX)

S1 Table Oligonucleotide primers used for qRT-PCR to amplify virus-specific and host-specific mRNAs. (DOCX)

## References

[1] Lamb RA, Krug RM. Orthomyxoviridae: the viruses and their replication. Fields Virology. 2001:1487–531.

[2] (WHO) WHO. Influenza (Seasonal) Fact sheet N°211. 2009.

[3] CDC. Inactivated influenza vaccine information statement. 2015.

[4] CDC. Prevention and control of influenza with vaccines: recommendations of the Advisory Committee on Immunization Practices (ACIP)—United States, 2012–13 influenza season. MMWR. 2012;61(32):613–8. 22895385

[5] Belshe RB, Newman FK, Wilkins K, Graham IL, Babusis E, Ewell M, et al Comparative immunogenicity of trivalent influenza vaccine administered by intradermal or intramuscular route in healthy adults. Vaccine. 2007;25(37–38):6755–63. 17692438 10.1016/j.vaccine.2007.06.066 PMC2148502

[6] Rimmelzwaan GF, Fouchier RAM, Osterhaus ADME. Influenza virus-specific cytotoxic T lymphocytes: a correlate of protection and a basis for vaccine development. Curr Opin Biotech. 2007;18(6):529–36. 18083548 10.1016/j.copbio.2007.11.002

[7] CDC. Live attenuated influenza vaccine [LAIV] (The nasal spray flu vaccine). 2015.

[8] Cox NJ, Kitame F, Kendal AP, Maassab HF, Naeve C. Identification of sequence changes in the cold-adapted, live attenuated influenza vaccine strain, A/Ann Arbor/6/60 (H2N2). Virology. 1988;167(2):554–67. 2974219

[9] Maassab HF. Adaptation and growth characteristics of influenza virus at 25°C. Nature. 1967;213(5076):612–4. 6040602 10.1038/213612a0

[10] Belshe RB, Ambrose CS, Yi T. Safety and efficacy of live attenuated influenza vaccine in children 2–7 years of age. Vaccine. 2008;26, Supplement 4:D10–D6. 18611422 10.1016/j.vaccine.2008.06.083

[11] Ambrose CS, Dubovsky F, Yi T, Belshe RB, Ashkenazi S. The safety and efficacy of live attenuated influenza vaccine in young children with asthma or prior wheezing. Eur J Clin Microbiol. 2012;31(10):2549–57. 10.1007/s10096-012-1595-9 PMC3456911 22410646

[12] Flannery B, Clippard J, Zimmerman RK, Nowalk MP, Jackson ML, Jackson LA, et al Early estimates of seasonal influenza vaccine effectiveness-United States, January 2015. MMWR. 2015;64(1):10–5. 25590680 PMC4584793

[13] Govorkova EA, McCullers JA. Therapeutics against influenza. Curr Top Microbiol Immunol. 2013;370:273–300. 10.1007/82_2011_198 22246228 PMC7121838

[14] Beigel J, Bray M. Current and future antiviral therapy of severe seasonal and avian influenza. Antivir Res. 2008;78(1):91–102. 10.1016/j.antiviral.2008.01.003 18328578 PMC2346583

[15] Baz M, Abed Y, Papenburg J, Bouhy X, Hamelin MÈ, Boivin G. Emergence of oseltamivir-resistant pandemic H1N1 virus during prophylaxis. New Engl J Med. 2009;361(23):2296–7. 10.1056/NEJMc0910060 19907034

[16] Mai LQ, Wertheim HFL, Duong TN, van Doorn HR, Hien NT, Horby P. A community cluster of oseltamivir-resistant cases of 2009 H1N1 influenza. New Engl J Med. 2010;362(1):86–7. 10.1056/NEJMc0910448 20007549

[17] Stephenson I, Democratis J, Lackenby A, McNally T, Smith J, Pareek M, et al Neuraminidase inhibitor resistance after oseltamivir treatment of acute influenza A and B in children. Clin Infect Dis. 2009;48(4):389–96. 10.1086/596311 19133796

[18] Moss WN, Priore SF, Turner DH. Identification of potential conserved RNA secondary structure throughout influenza A coding regions. RNA. 2011;17(6):991–1011. 10.1261/rna.2619511 21536710 PMC3096049

[19] Gultyaev AP, Heus HA, Olsthoorn RC. An RNA conformational shift in recent H5N1 influenza A viruses. Bioinformatics. 2007;23(3):272–6. 17090581 10.1093/bioinformatics/btl559

[20] Jiang T, Kennedy SD, Moss WN, Kierzek E, Turner DH. Secondary structure of a conserved domain in an intron of influenza A M1 mRNA. Biochemistry. 2014;53(32):5236–48. 10.1021/bi500611j 25026548 PMC4139153

[21] Moss WN, Dela-Moss LI, Kierzek E, Kierzek R, Priore SF, Turner DH. The 3' splice site of influenza A segment 7 mRNA can exist in two conformations: a pseudoknot and a hairpin. PLoS One. 2012;7(6):e38323 10.1371/journal.pone.0038323 22685560 PMC3369869

[22] Zadeh JN, Wolfe BR, Pierce NA. Nucleic acid sequence design via efficient ensemble defect optimization. J Comput Chem. 2011;32(3):439–52. 10.1002/jcc.21633 20717905

[23] Landau L, Lifshitz E, Reichl LE. Statistical Physics Part 1, 3rd ed Butterworth-Heinemann: New York 1980.

[24] Mathews DH. Using an RNA secondary structure partition function to determine confidence in base pairs predicted by free energy minimization. RNA. 2004;10(8):1178–90. 15272118 10.1261/rna.7650904 PMC1370608

[25] Reuter J, Mathews D. RNAstructure: software for RNA secondary structure prediction and analysis. BMC Bioinformatics. 2010;11(1):129. 20230624 10.1186/1471-2105-11-129 PMC2984261

[26] Hajdin CE, Bellaousov S, Huggins W, Leonard CW, Mathews DH, Weeks KM. Accurate SHAPE-directed RNA secondary structure modeling, including pseudoknots. Proc Natl Acad Sci USA. 2013;110(14):5498–503. 10.1073/pnas.1219988110 23503844 PMC3619282

[27] Schickli J, Flandorfer A, Nakaya T, Martinez-Sobrido L, Garcia-Sastre A, Palese P. Plasmid-only rescue of influenza A virus vaccine candidates. Philos Trans R Soc Lond B Biol Sci. 2001;356:1965–74. 11779399 10.1098/rstb.2001.0979 PMC1088576

[28] Martinez-Sobrido L, Garcia-Sastre A. Generation of recombinant influenza virus from plasmid DNA. J Visualized Experiments. 2010(42). 10.3791/2057 PMC3156010 20729804

[29] Baker SF, Guo H, Albrecht RA, Garcia-Sastre A, Topham DJ, Martinez-Sobrido L. Protection against lethal influenza with a viral mimic. J Virol. 2013;87(15):8591–605. 10.1128/JVI.01081-13 23720727 PMC3719819

[30] Pfaffl MW. A new mathematical model for relative quantification in real-time RT–PCR. Nucl Acids Res. 2001;29(9):e45–e. 11328886 10.1093/nar/29.9.e45 PMC55695

[31] Gu M, Huang J, Chen Y, Chen J, Wang X, Liu X, et al Genome sequence of a natural reassortant H5N2 avian influenza virus from domestic mallard ducks in Eastern China. J Virol. 2012;86(22):12463–4. 10.1128/JVI.02315-12 23087121 PMC3486508

[32] Chen JL, Dishler AL, Kennedy SD, Yildirim I, Liu B, Turner DH, et al Testing the nearest neighbor model for canonical RNA base pairs: revision of GU parameters. Biochemistry. 2012;51(16):3508–22. 10.1021/bi3002709 22490167 PMC3335265

[33] Tobita K, Sugiura A, Enomoto C, Furuyama M. Plaque assay and primary isolation of influenza A viruses in an established line of canine kidney cells (MDCK) in the presence of trypsin. Med Microbiol Immun. 1975;162(1):9–14. 10.1007/BF02123572 1214709

[34] Tyagi R, Mathews DH. Predicting helical coaxial stacking in RNA multibranch loops. RNA. 2007;13(7):939–51. 17507661 10.1261/rna.305307 PMC1894924

[35] Walter AE, Turner DH. Sequence dependence of stability for coaxial stacking of RNA helixes with Watson-Crick base paired interfaces. Biochemistry. 1994;33(42):12715–9. 7522562 10.1021/bi00208a024

[36] Walter AE, Turner DH, Kim J, Lyttle MH, Müller P, Mathews DH, et al Coaxial stacking of helixes enhances binding of oligoribonucleotides and improves predictions of RNA folding. Proc Natl Acad Sci USA. 1994;91(20):9218–22. 7524072 10.1073/pnas.91.20.9218 PMC44783

[37] Gultyaev AP, Tsyganov-Bodounov A, Spronken MI, van der Kooij S, Fouchier RA, Olsthoorn RC. RNA structural constraints in the evolution of the influenza A virus genome NP segment. RNA Biol. 2014;11(7):942–52. 10.4161/rna.29730 25180940 PMC4179967

[38] Schnell JR, Chou JJ. Structure and mechanism of the M2 proton channel of influenza A virus. Nature. 2008;451(7178):591–5. 10.1038/nature06531 18235503 PMC3108054

[39] Arzt S, Baudin F, Barge A, Timmins P, Burmeister WP, Ruigrok RWH. Combined results from solution studies on intact influenza virus M1 protein and from a new crystal form of its N-terminal domain show that M1 is an elongated monomer. Virology. 2001;279(2):439–46. 11162800 10.1006/viro.2000.0727

[40] Lommer BS, Luo M. Structural plasticity in influenza virus protein NS2 (NEP). J Biol Chem. 2002;277(9):7108–17. 11751904 10.1074/jbc.M109045200

[41] Zhang K, Wang Z, Liu X, Yin C, Basit Z, Xia B, et al Dissection of influenza A virus M1 protein: pH-dependent oligomerization of N-terminal domain and dimerization of C-terminal domain. PLoS One. 2012;7(5):e37786 10.1371/journal.pone.0037786 22655068 PMC3360003

[42] Safo MK, Musayev FN, Mosier PD, Zhou Q, Xie H, Desai UR. Crystal structures of influenza A virus matrix protein M1: variations on a theme. PLoS One. 2014;9(10):e109510 10.1371/journal.pone.0109510 25295515 PMC4190115

[43] Shtykova EV, Baratova LA, Fedorova NV, Radyukhin VA, Ksenofontov AL, Volkov VV, et al Structural analysis of influenza A virus matrix protein M1 and its self-assemblies at low pH. PLoS One. 2013;8(12):e82431 10.1371/journal.pone.0082431 24358182 PMC3865061

[44] Cross TA, Dong H, Sharma M, Busath DD, Zhou H-X. M2 protein from Influenza A: from multiple structures to biophysical and functional insights. Curr Opin Virol. 2012;2(2):128–33. 10.1016/j.coviro.2012.01.005 22482709 PMC3322387

[45] Holsinger LJ, Nichani D, Pinto LH, Lamb RA. Influenza A virus M2 ion channel protein: a structure-function analysis. J Virol. 1994;68(3):1551–63. 7508997 10.1128/jvi.68.3.1551-1563.1994 PMC236612

[46] Cho KJ, Schepens B, Seok JH, Kim S, Roose K, Lee JH, et al Structure of the extracellular domain of matrix protein 2 of influenza A virus in complex with a protective monoclonal antibody. J Virol. 2015;89(7):3700–11. 10.1128/JVI.02576-14 25609808 PMC4403401

[47] Bornholdt ZA, Prasad BVV. X-ray structure of influenza virus NS1 effector domain. Nat Struct Mol Biol. 2006;13(6):559–60. 16715094 10.1038/nsmb1099

[48] Carrillo B, Choi J-M, Bornholdt ZA, Sankaran B, Rice AP, Prasad BV. The influenza A virus protein NS1 displays structural polymorphism. J Virol. 2014;88(8):4113–22. 10.1128/JVI.03692-13 24478439 PMC3993732

[49] Cheng A, Wong SM, Yuan YA. Structural basis for dsRNA recognition by NS1 protein of influenza A virus. Cell Res. 2009;19(2):187–95. 10.1038/cr.2008.288 18813227

[50] Hale BG, Kerry PS, Jackson D, Precious BL, Gray A, Killip MJ, et al Structural insights into phosphoinositide 3-kinase activation by the influenza A virus NS1 protein. Proc Natl Acad Sci USA. 2010;107(5):1954–9. 10.1073/pnas.0910715107 20133840 PMC2808220

[51] Das K, Ma L-C, Xiao R, Radvansky B, Aramini J, Zhao L, et al Structural basis for suppression of a host antiviral response by influenza A virus. Proc Natl Acad Sci USA. 2008;105(35):13093–8. 10.1073/pnas.0805213105 18725644 PMC2522260

[52] Pace CN, Scholtz JM. A helix propensity scale based on experimental studies of peptides and proteins. Biophys J. 1998;75(1):422–7. 9649402 10.1016/s0006-3495(98)77529-0 PMC1299714

[53] Snyder M, Betts R, DeBorde D, Tierney E, Clements M, Herrington D, et al Four viral genes independently contribute to attenuation of live influenza A/Ann Arbor/6/60 (H2N2) cold-adapted reassortant virus vaccines. J Virol. 1988;62(2):488–95. 3336068 10.1128/jvi.62.2.488-495.1988 PMC250559

[54] Osterholm MT, Kelley NS, Sommer A, Belongia EA. Efficacy and effectiveness of influenza vaccines: a systematic review and meta-analysis. Lancet Infect Dis. 2012;12(1):36–44. 10.1016/S1473-3099(11)70295-X 22032844

[55] De Villiers PJ, Steele AD, Hiemstra LA, Rappaport R, Dunning AJ, Gruber WC, et al Efficacy and safety of a live attenuated influenza vaccine in adults 60 years of age and older. Vaccine. 2009;28(1):228–34. 10.1016/j.vaccine.2009.09.092 19796721

[56] Cox A, Baker SF, Nogales A, Martinez-Sobrido L, Dewhurst S. Development of a mouse-adapted live attenuated influenza virus that permits in vivo analysis of enhancements to the safety of live attenuated influenza virus vaccine. J Virol. 2015;89(6):3421–6. 10.1128/JVI.02636-14 25552727 PMC4337518

