# Supplementary material for: Mutations Designed by Ensemble Defect to Misfold Conserved RNA Structures of Influenza A Segments 7 and 8 Affect Splicing and Attenuate Viral Replication in Cell Culture
Source: PLoS One. 2016 Jun 7;11(6):e0156906. doi: 10.1371/journal.pone.0156906 (PMC4896458; doi:10.1371/journal.pone.0156906)
Supplement: S1 Fig — (DOCX) [file pone.0156906.s001.docx]

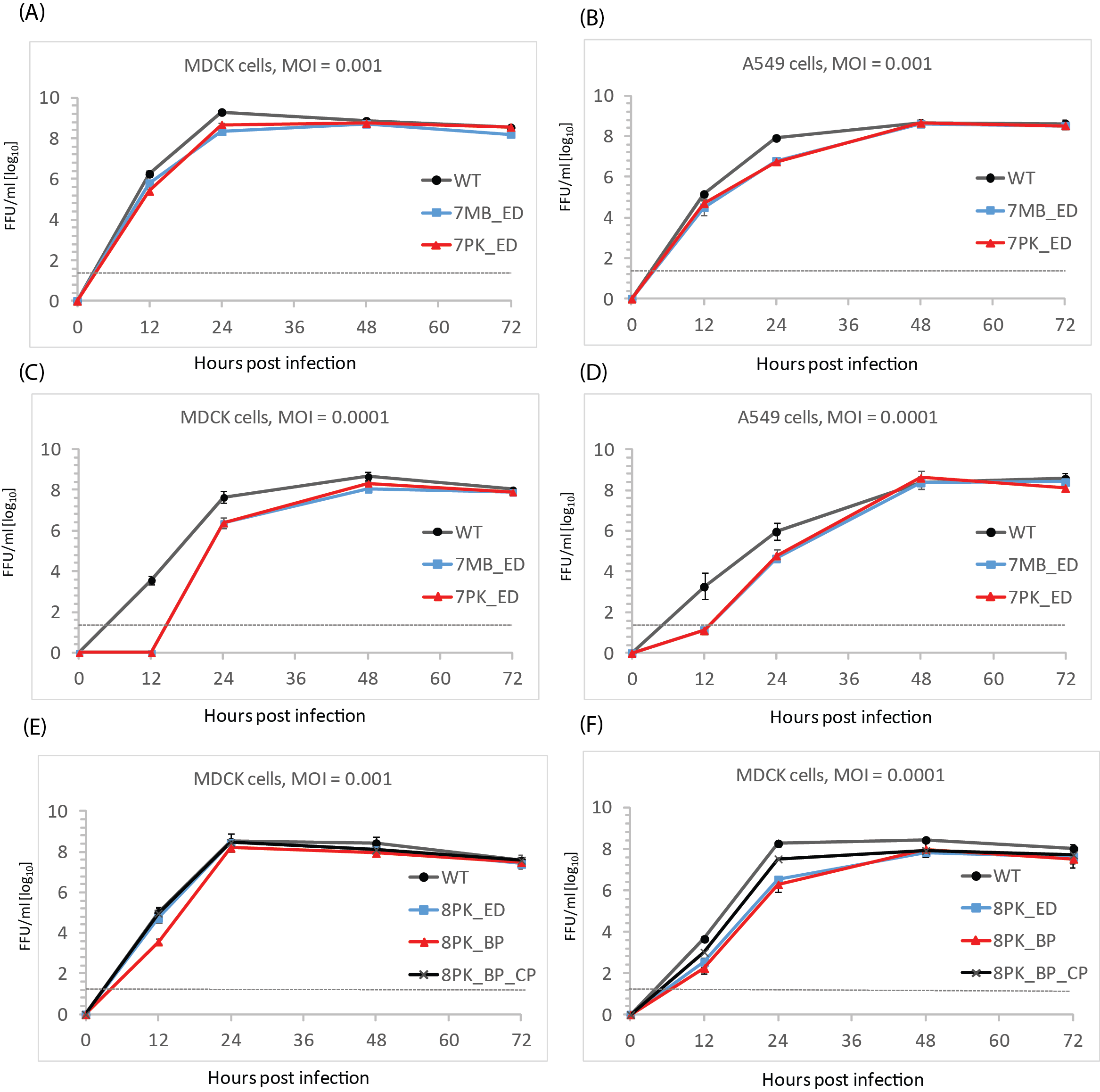


**S1 Figure. Multi-cycle growth kinetics of WT and mutant viruses (Fig. 4) plotted in log-scale.** Virus titers were quantified by immunofocus assay (FFU/ml). Data represent the means ± SDs of the results determined for triplicate wells. Replication of WT, 7MB_ED and 7PK_ED were examined in (A) MDCK cells and (B) A549 cells with MOI = 0.001, and in (C) MDCK cells and (D) A549 cells with MOI = 0.0001. Replication of WT, 8PK_ED, 8PK_BP, and 8PK_BP_CP were examined in MDCK cells with (E) MOI = 0.001 and (F) MOI = 0.0001.
