# Supplementary material for: Mutations Designed by Ensemble Defect to Misfold Conserved RNA Structures of Influenza A Segments 7 and 8 Affect Splicing and Attenuate Viral Replication in Cell Culture
Source: PLoS One. 2016 Jun 7;11(6):e0156906. doi: 10.1371/journal.pone.0156906 (PMC4896458; doi:10.1371/journal.pone.0156906)
Supplement: S1 Table — (DOCX) [file pone.0156906.s002.docx]

| **mRNA amplified** | **primer sets** | **primer sequences** |
| --- | --- | --- |
| M1 | sense | GTGACAACAACCAATCCA |
|  | antisense | CTGACTAGCAACCTCCAT |
| M2 | sense | GAGGTCGAAACGCCTATC |
|  | antisense | AGACGATCAAGAATCCACAA |
| NS1 | sense | GACCAAGAACTAGGTGATGC |
|  | antisense | CGCTCCACTATCTGCTTTCC |
| NEP | sense | CTTTCAGGACATACTGCT |
|  | antisense | CGAACTGTGTTATCATTCC |
| GAPDH  (MDCK cells) | sense | AATGTATCAGTTGTGGATCT |
|  | antisense | TGCTTCACTACCTTCTTG |
| GAPDH  (A549 cells) | sense | CTCATGACCACAGTCCATGC |
|  | antisense | TTCAGCTCAGGGATGACCTT |

**S1 Table. Oligonucleotide primers used for qRT-PCR to amplify virus-specific and host-specific mRNAs.** Primers were synthesized by Integrated DNA Technologies.
